# Tuberculosis and cardiovascular disease: linking the epidemics

**DOI:** 10.1186/s40794-015-0014-5

**Published:** 2015-10-30

**Authors:** Moises A. Huaman, David Henson, Eduardo Ticona, Timothy R. Sterling, Beth A. Garvy

**Affiliations:** 1 Division of Infectious Diseases, Department of Medicine, University of Kentucky, 40536 Lexington, KY USA; 2 Infectious Diseases & Tropical Medicine Research Unit, Hospital Nacional Dos de Mayo, Lima, Peru; 3 Division of Infectious Diseases, Department of Medicine, Vanderbilt University, Nashville, TN USA; 4 Department of Microbiology, Immunology and Molecular Genetics, University of Kentucky, Lexington, KY USA

**Keywords:** Tuberculosis, Cardiovascular diseases, Atherosclerosis, Inflammation, Epidemics, Communicable disease

## Abstract

The burden of tuberculosis and cardiovascular disease (CVD) is enormous worldwide. CVD rates are rapidly increasing in low- and middle-income countries. Public health programs have been challenged with the overlapping tuberculosis and CVD epidemics. Monocyte/macrophages, lymphocytes and cytokines involved in cellular mediated immune responses against *Mycobacterium tuberculosis* are also main drivers of atherogenesis, suggesting a potential pathogenic role of tuberculosis in CVD via mechanisms that have been described for other pathogens that establish chronic infection and latency. Studies have shown a pro-atherogenic effect of antibody-mediated responses against mycobacterial heat shock protein-65 through cross reaction with self-antigens in human vessels. Furthermore, subsets of mycobacteria actively replicate during latent tuberculosis infection (LTBI), and recent studies suggest that LTBI is associated with persistent chronic inflammation that may lead to CVD. Recent epidemiologic work has shown that the risk of CVD in persons who develop tuberculosis is higher than in persons without a history of tuberculosis, even several years after recovery from tuberculosis. Together, these data suggest that tuberculosis may play a role in the pathogenesis of CVD. Further research to investigate a potential link between tuberculosis and CVD is warranted.

## Background

Non-communicable diseases caused 38 of the 56 million deaths reported globally in 2012. About half of these deaths were related to cardiovascular disease (CVD) [1]. CVD is expected to be the major cause of death for most developing countries by 2020, similar to the current epidemiology of CVD in developed nations [2, 3]. Compared to high-income countries, CVD occurs at a younger age and causes a larger proportion of premature deaths in low- and middle-income countries [4]. Traditional risk factors such as obesity, hypertension and diabetes explain much of this increase [4], but studies indicate that the burden of infection may also contribute to the development of CVD [5].

Over 2 billion persons are infected with *Mycobacterium tuberculosis* globally and about 2 million die from tuberculosis disease every year [6]. Tuberculosis and non-communicable diseases may not only co-exist, but also augment the risk of each other [7]. This review seeks to explore the association between tuberculosis and CVD.

## Review

### A connection between infection and cardiovascular disease

The hypothesis that infection has a pathogenic role in CVD was first suggested by clinical observations. The first convincing evidence of a connection between infection and CVD arose from the work by Fabricant et al. more than 3 decades ago [8]. This group showed that infection of chickens with Marek’s disease virus, an avian herpesvirus, caused atherosclerotic lesions in coronary arteries and other vessels [9]. In 1992, Shor et al. in South Africa found *Chlamydia pneumoniae* in the fatty streaks of coronary artery plaques [10]. Subsequent experiments in animal models supported a pathogenic role of *C. pneumoniae* in atheroma formation [11–13]. Serologic studies followed and showed an association between *C. pneumoniae* antibodies and CVD in humans [14]. Similarly, *Helicobacter pylori* exists in atherosclerotic plaques, and elimination of the infection increases the coronary artery lumen and reduces cardiac event rates [15, 16]. Influenza virus is associated with acute myocardial infarction (AMI) [17], as well as accelerated development of early coronary artery plaques [18, 19]. Human immunodeficiency virus (HIV) has also been linked to CVD. HIV infection increases the risk of cardiovascular events 1.5 – 2-fold after adjusting for traditional CVD risk factors [20]. This appears to be mediated at least in part by chronic immune activation caused by HIV, even after suppressing the virus with antiretroviral therapy [21]. Infections due to hepatitis B virus [22], hepatitis C [23], Epstein Barr virus [24, 25], cytomegalovirus (CMV) [26, 27] and periodontal bacteria [28] have also been associated with atherosclerosis and CVD through chronic systemic inflammation and other mechanisms. Notably, most of the pathogens implicated in CVD pathogenesis are intracellular organisms and/or may be able to establish chronic or latent infection in humans [29]. This may cause persistent local or systemic inflammation that can lead to atherosclerotic plaque formation. Recent studies suggest that latent tuberculosis infection (LTBI) is associated with chronic inflammation therefore a connection between LTBI and CVD seems plausible [30, 31].

The effect of acute infection on short-term cardiovascular events has also been studied. Acute lower respiratory tract infections carry a high risk of subsequent AMI or stroke. A large population-based retrospective study conducted in the United Kingdom showed a 5-fold increase of AMI in patients diagnosed with an acute systemic respiratory infection within the prior 3 days. Furthermore, the risk of AMI remained elevated 1 to 3 months after the infection [32]. *M. tuberculosis* primarily causes pulmonary disease. Although sub-acute or chronic presentation is common, acute illness from tuberculosis can also occur [33]. A recent report showed that persons with pulmonary or extrapulmonary tuberculosis had an increased subsequent risk of AMI and unstable angina. Surprisingly, the risk remained elevated after several years from the initial diagnosis of tuberculosis suggesting that tuberculosis disease may have short-term and long-term implications in CVD [34]. Alternatively, developing tuberculosis disease may just be a marker of background dysfunctional immune responses in susceptible hosts, as these same abnormal responses that predispose to tuberculosis may also predispose to CVD.

### Mechanisms of the effect of infection on cardiovascular disease

Two main themes dominate the proposed mechanism that connects infection and CVD; 1) an increase in inflammation leading to coronary artery plaque formation and/or plaque rupture, and 2) autoimmune disease. An additional hypothesis is that pathogens inhabit growing atherosclerotic plaques, and cause direct vascular damage [5].

For an infectious disease to cause CVD via an increase in inflammation it stands to reason that the infection must trigger host immunologic responses similar to those implicated in atherogenesis. Plaques begin as a small inflammatory process seen as reversible fatty streaks in the innermost part of the artery [35]. Endothelial cells are activated by released phospholipids and other stimuli, and this activation increases surface adhesion molecules (i.e.; intracellular adhesion molecule-1, ICAM-1) that bind immune cells including circulating monocytes, lymphocytes and others [36]. These immune cells access the intima and accelerate plaque growth [37]. CD4^+^ cells of the T helper 1 (TH1) type play a key role in atherogenesis. The production of interferon (IFN)-γ, tumor necrosis factor (TNF)-α and interleukin (IL)-1 by these CD4^+^ TH1 cells contribute to growth of the plaque, as does macrophage colony stimulating factor produced by endothelial cells [38, 39]. These chemokines activate monocyte/macrophages and other immune cells in a positive feedback loop [39]. Studies have shown the important role of IFN-γ, particularly in atherogenesis [40]. Mice without IFN-γ signaling develop plaque at a substantially lower rate compared to mice with IFN-γ signaling [41]. As these plaques progress through stages of development they become covered by a cap of smooth-muscle cells and collagen-rich matrix. Some plaques may include calcifications and thrombi [35]. The most vulnerable part of the plaque, the cap, may rupture when triggered by a variety of stresses. Plaque rupture causes 60 % to 70 % of coronary artery thrombotic events [42].

Inflammation related to infection can augment many of these steps, from early plaque formation to ultimate plaque rupture. Furthermore, pathogens may not need to be fully proliferating to cause inflammation [5]. For instance, a study of the mechanism of Epstein Barr virus (EBV) in CVD showed that the presence of neutralizing antibodies to an early EBV protein was associated with higher levels of IL-6 and ICAM-1 in patients with AMI compared to patients with stable angina [25]. This protein, deoxyuridine triphosphate nucleotidohyrolase, is an early EBV product expressed during lytic and abortive-lytic EBV replication that leads to NF-κβ activation through toll like receptor 2 [43]. Thus even under conditions of incomplete viral replication EBV may have a role in precipitating AMI [25]. Similarly, studies indicate that non-replicating CMV may stimulate the host immune system and promote atherogenesis [44].

The most common cross reaction of antibodies from infection to self-proteins in atherosclerosis centers on the heat shock protein (HSP) system [45]. The HSP system has been strongly conserved throughout evolution. The sequence of human HSP60 has 40–50 % identical residues, and 20 % conservative replacements to HSP65 in *Mycobacterium* spp. and HSP60 in *Escherichia coli* [46]. This conservation of epitopes results in a cross-reaction between antibodies produced to bacterial HSP such as *E. coli* HSP60, chlamydial HSP60 or mycobacterial HSP65, and the self HSP60 in humans. Perschinka et al. showed that antibodies against bacterial HSP60/65 also bound the homologues epitopes on human HSP60 [47]. A study showed that antibodies to HSP65 were the only antibodies significantly tied to CVD [48]. This is critically important because endothelial cells express HSP on their surface when they are activated by infection or other stressors [48]. This expression provides a target for the immune reaction to HSP, which can lead to plaque development. A heightened level of anti-HSP65 antibody was found in individuals with elevated coronary calcification scores, after controlling for other cardiac risk factors [49].

### Evidence connecting tuberculosis disease and cardiovascular disease

The hypothesis that tuberculosis leads to CVD comes from case studies of tuberculosis causing cardiovascular death as well as from population based studies that show increased risk of cardiovascular events.

Tuberculous granuloma formation affecting the coronary arteries has been described as a rare cause of myocardial infarction in young patients [50, 51]. The idea that infection could predispose to CVD early in life is supported by work by Dummer et al. who found a correlation between CMV infection and coronary atherosclerosis of explanted hearts in young patients who underwent heart transplantation, but not among older individuals [52]. It is possible that tuberculosis could be contributing to premature cardiovascular death in areas of high tuberculosis prevalence.

*M. tuberculosis* may not only affect the coronary vessels, but also the myocardium. Liu et al. summarized available clinical cases of tuberculous myocarditis and related sudden cardiac death (SCD) [53]. Most patients were young and asymptomatic prior to their fatal event. The proposed underlying mechanism leading to SCD was ventricular tachyarrhythmia, likely a consequence of aberrant conduction from extensive tuberculous septal involvement or ventricular wall necrosis [54, 55]. Pulmonary tuberculosis without obvious direct mycobacterial involvement of the myocardium or coronary arteries has also been liked to SCD, but the exact mechanisms of death remain obscure [56].

A large population-based retrospective cohort study conducted in Taiwan goes beyond these individual clinical observations to look at baseline CVD risk elevation in patients who had tuberculosis disease. The researchers looked at 10,168 patients with a history of tuberculosis disease and 40,672 control patients without a history of tuberculosis disease. After adjusting for important co-morbidities, the tuberculosis group had a 40 % increased risk of the composite endpoint of unstable angina and AMI compared to the non-tuberculosis group. This elevated risk persisted for the entire study period of up to 14 years [34].

The potential effects of tuberculosis disease do not appear to be limited to coronary heart disease (CHD) but extend to other atherosclerosis-mediated vascular diseases such as stroke. A study in Taiwan followed patients with a history non-meningeal tuberculosis disease and no history of stroke, as well as control patients. The authors found a 50 % increased risk of ischemic stroke in the tuberculosis group after 3 years of follow-up. This study also showed an increased risk of CHD in the tuberculosis group [57].

### Mechanism of cardiovascular disease in tuberculosis disease

The possible mechanisms of CVD in tuberculosis are summarized in the Table 1. In tuberculous myocarditis or coronary arteritis the relationship between infection and CVD is direct and apparent. However, these extrapulmonary complications of *M. tuberculosis* disease seem rare [53]. As in other infections, the role of chronic inflammation and HSP-based autoimmunity may have consequences in CVD at a much larger scale.

**Table 1:** Possible mechanisms of cardiovascular disease in tuberculosis

Direct effect on the myocardium (tuberculous myocarditis)
Direct effect on coronary arteries (tuberculous arteritis)
Increased expression of pro-inflammatory citokines (i.e., IL-1, IL-2, IL-6, IFN-γ, TNF-α)
Monocyte/macrophage immune activation
CD4^+^ TH1 and TH17 cell immune activation
Auto-immunity mediated by antibodies against mycobacterial HSP65

Abreviations: *HSP65* heat shock protein 65, *IL* interleukin, *IFN* interferon, *TH1 T helper 1*, *TH17*, T helper 17, *TNF* tumor necrosis factor

In tuberculosis, the innate immune system initiates an immune response within the lung. This response deploys large numbers of phagocytic cells, which also play host to *Mycobacterium tuberculosis* [58, 59]. As the reaction progresses, immune cells continue to accumulate in the lungs so that more host cells become available to the bacteria, and eventually a granuloma forms. Granuloma formation relies on inflammatory chemical mediators to bring other immune cells to the site of infection [60]. After the initial innate immune reaction, immunological equilibrium develops via an adaptive response. Once the adaptive response is established, CD4^+^ T cells become activated and release a plethora of cytokines to modulate the immune response against *M. tuberculosis* [61]. Similar to inflammatory responses related to CVD, TNF-α and IFN-γ are of particular importance. Blockage of either leads to severe mycobacterial infections [62–64]. Patients with tuberculosis disease also have a significant elevation in IL-1, IL-2, IL-6 and IL-22. The total level of T cells is decreased, but those present shift strongly to a TH1 type. When stimulated with mycobacterial antigens, they readily produce IL-17, IL-22 and IFN-γ [30]. This TH1 inflammation matches the inflammatory profile of plaque formation presented earlier. Thus, tuberculosis may have a pathogenic role in CVD through activation of cell-mediated immune responses that promote atherogenesis.

Emerging evidence indicates a role for IL-17 and the CD4^+^ subset T helper 17 (TH17) cells in immune responses to tuberculosis [65]. Early in the immune response to tuberculosis IL-17 production is dominated by γδ cells [66]. IL-17 is required for the development of a mature granuloma possibly through an increase in cell adhesion molecules [67]. In long term immune response the production of IL-17 is maintained by antigen specific TH17 cells, and without this response antimicrobial activity in the lungs is reduced [68]. IL-17 deficient mice show an impaired response to virulent tuberculosis [67].

In atherosclerosis, TH17 cells have been regarded both protective and pathogenic roles [69]. Pathogenic TH17 cells are induced by IL-6, IL-23 and IL-1β; and produce IL-17 and IFN- γ [70, 71]. Pathogenic TH17 cells have been found in atherosclerotic plaques of mice and humans, and the presence of both IL-17 and IFN- γ have a synergistic pro-inflammatory effect on vascular smooth muscle cells [72]. It is possible that TH17 cells implicated in immune responses to tuberculosis may have a role in atherogenesis.

Studies show that mycobacterial HSP65 share homology to human HSP60 [46]. Antibodies specific to the entire mycobacterial HSP65 cross-react with human HSP60. When antibodies are targeted to specific epitopes of mycobacterial HSP65, cross reactivity remains [47]. Injection of mycobacterial HSP65 protein induces arteriosclerosis in rabbits with both normal and elevated cholesterol levels, unlike any other control antigen [73]. In humans, a significant correlation between anti-HSP65 antibodies and carotid atherosclerosis has been reported [48, 49]. These data suggest that tuberculosis could have a pathogenic role in CVD through molecular mimicry and auto-immunity mediated by anti-HSP65 antibodies.

### Tuberculosis and Takayasu’s arteritis

A connection between tuberculosis and Takayasu’s arteritis (TA) has been suggested. TA is a chronic vasculitis of unknown origin that primarily affects the aorta and its main branches. The diagnosis and treatment of TA was recently reviewed by Isobek [74].

In 1981 Pantell et al. presented a compilation of case series from Japan, Korea, India, Mexico and South Africa that included 747 cases of TA. The rates of tuberculosis disease in these case series ranged from 22 % to 70 %, compared to tuberculosis rates of 0.03 % to 1.5 % for the general population in those countries [75]. This report and others that followed suggested an association between tuberculosis disease and TA [76]; however, further investigation is clearly needed. Aggarwal et al. looked at antibody titers against *M. tuberculosis* proteins including HSP65 in TA. The authors found a significant increase in immunoglobulin (Ig) G, IgM, and IgA titers to *M. tuberculosis* antigens; and IgM and IgA titers against HSP65 in patients with TA compared to control patients without TA. Although this would further support a potential link between tuberculosis and TA, the authors postulated that the observed antibody responses could also be secondary to prior Bacillus Calmette-Guerin (BCG) vaccination [77].

### Latent tuberculosis infection and cardiovascular disease

A link between LTBI and CVD via chronic inflammation seems biologically plausible. The classic model of LTBI stipulates that mycobacteria in the inflammatory capsule do not divide but remain in stasis, enduring the environment unsuitable for growth. The constant level of colony forming units isolated from LTBI lesions indicated this lack of division. Researchers supported this model by testing the count of *M. tuberculosis* within the tubercles by using rtPCR and showed that the level of genetic material within a tubercle does not change overtime [78]. The first indication that this picture might be incomplete came from the clinical experience with isoniazid. This antibiotic is active against LTBI but only shows effectiveness against dividing mycobacterium both in vitro and in vivo [79, 80]. Therefore if isoniazid works in LTBI then mycobacterial division would be expected to occur in the latent form. Gill et al. demonstrated that there is continued replication of mycobacterium in chronically infected mice. This group inserted an unstable plasmid into *M. tuberculosis* and showed that tissue levels of the plasmid decreased with every division of the bacterium. Because the total number of bacteria remained constant the authors concluded that bacteria that die must be cleared away from the site by the host immune system therefore giving a false impression of absent replication [81]. These data suggests that subsets of mycobacteria are actively dividing in LTBI, and a dynamic equilibrium between the host immune system and the bacteria is established during latency. In this context, a degree of chronic inflammation driven by LTBI is possible. Furthermore, studies in cynomolgus macaques infected with latent tuberculosis have shown that each granuloma within a single host possesses a unique profile of T cells and pro- and anti-inflammatory cytokines leading to a spectrum of granulomas with differential mycobacterial killing capacity within the same host at any given time, demonstrating that immune responses and bacterial burden in LTBI are quite dynamic [82].

A study of immune activation in patients with latent and active tuberculosis in Canada showed an inflammatory profile consistent with this model. The baseline circulating levels of TNF-α, IL-1β, IL-4, IL-8, and IL-22 were all significantly elevated in patients with LTBI compared to healthy controls with a negative tuberculin skin test (TST). IFN-γ levels were also higher in the LTBI group compared to healthy controls but the difference was not statistically significant [30]. Similarly, a study of HIV-infected persons in South Africa showed elevated levels of circulating soluble CD14, C-reactive protein, IL-6 and interferon gamma-induced protein 10 (IP10) among patients with HIV and LTBI co-infection, compared to HIV patients without evidence of tuberculosis infection based on a negative TST, negative IFN- γ ELISpot, negative chest-X-ray, negative induced sputum for *M. tuberculosis* and no tuberculosis disease symptoms [31].

A recent study looked at markers of systemic inflammation in persons with tuberculosis disease, LTBI, prior tuberculosis, and healthy controls in India. Compared to healthy controls, patients with LTBI were found to have higher levels of monocyte/macrophage activation and inflammatory mediators including CD14, CXCL3, CCL2 and CCL8. This suggests a switch from neutrophil to monocyte/macrophage centered responses in these patients. The authors concluded that LTBI may induce long-term persistent inflammation, even in the absence of tuberculosis disease [83]. Interestingly, these researchers also found similar monocyte/macrophage activation markers in persons cured from prior tuberculosis, which may explain the increased risk for AMI and unstable angina observed years after recovery from tuberculosis in the Taiwan cohort study discussed above [34].

Not all studies that looked at LTBI and inflammatory markers found a positive association. For instance, decreased IL-17 expression and increased T regulatory cell activity has been described in the context of LTBI [84]. Wergeland et al. showed that CD4^+^ T cell activation in LTBI is lower than in tuberculosis disease. However, a wide range of CD4^+^ T cell activation was present in persons with LTBI [85]. The authors postulate that this could be an indication of differential immune activation throughout the stages of tuberculosis infection and related mycobacterial burden. This model would be consistent with current evidence that redefines LTBI as a dynamic spectrum rather than a homogeneous state [86]. Further research is needed to define the potential role of LTBI in CVD, as this could have important implications worldwide.

## Conclusions

With increasing evidence that many infections contribute to the pathogenesis of CVD, the potential role of tuberculosis in CVD is not surprising. A potential mechanistic model for this association is based on research showing persistent immune activation in latent and active tuberculosis. Antibodies to mycobacterial HSP65 cross-reacting with self-antigens in human vessels leading to autoimmunity may also have an effect on CVD risk. Further research to investigate the potential link between tuberculosis and CVD is warranted.

## Abbreviations

AMI: Acute myocardial infarction
BCG: Bacillus Calmette-Guerin
CAD: Cardiovascular disease
CHD: Coronary heart disease
CMV: Cytomegalovirus
EBV: Epstein Barr virus
HIV: Human immunodeficiency virus
HSP: Heat shock protein
ICAM-1: Intracellular adhesion molecule-1
IFN: Interferon
Ig: Immunoglobulin
IL: Interleukin
IP10: Interferon gamma-induced protein 10
LTBI: Latent tuberculosis infection
SCD: Sudden Cardiovascular Death
TA: Takayasu’s arteritis
TH1: T helper 1
TH17: T helper 17
TNF: Tumor necrosis factor
TST: Tuberculin skin test
